# Inflammation in Health and Disease: New Insights and Therapeutic Avenues

**DOI:** 10.3390/ijms23158392

**Published:** 2022-07-29

**Authors:** Morena Scotece, Javier Conde-Aranda

**Affiliations:** 1 Molecular Mechanisms of Cancer Program, Centro de Investigación del Cáncer (CIC), Instituto de Biología Molecular y Celular del Cáncer (IBMCC), CSIC-USAL, 37007 Salamanca, Spain; morena.scotece84@gmail.com; 2 Molecular and Cellular Gastroenterology, Health Research Institute of Santiago de Compostela (IDIS), 15706 Santiago de Compostela, Spain

The inflammatory response is an adaptive mechanism that evolved to fight against infections and tissue damage. This complex mechanism is vital for the proper function of the organism, and because of that, is under tight regulation involving a complex network of factors, cells, and systems. Physiological inflammation is perfectly orchestrated to allow the mobilization of leukocytes from the circulation to the injured tissues for the removal of pathogens, tissue repair, and return to homeostasis. However, when the self-limited nature of the inflammatory response and the mechanisms of resolution fail, inflammation might become chronic, leading to the development of many disabling serious diseases such as rheumatoid arthritis (RA), inflammatory bowel disease (IBD), or psoriasis.

Knowledge of the molecular and cellular processes underlying chronic inflammation has substantially increased in recent decades. This resulted in enormous improvements in the treatments for immune-mediated inflammatory diseases, but also revealed the complexity of the cytokine networks and immune cell subtypes involved in non-resolving inflammatory responses. This fact could explain, in part, why certain groups of patients are totally or partially refractory to current therapies. Therefore, we cannot be satisfied with the available drugs, and more research focusing on deciphering the precise molecular pathways behind uncontrolled inflammation is mandatory for developing novel therapeutic approaches.

The 34 original and review articles included in this Special Issue contributed to the aim of describing and understanding new mechanisms that trigger inflammation and highlighted new putative treatments for different inflammatory conditions.

Neuroinflammation is a perfect example of the two faces of inflammation. While an acute inflammatory response is protective, chronic neuroinflammation leads to inflammatory conditions such as multiple sclerosis, and it also contributes to other neurological [1] and psychiatric disorders such as Alzheimer’s or Parkinson’s disease [2]. Microglia is pivotal for regulating central nervous system inflammation. Therefore, it would be interesting to develop therapeutic strategies conducted to mitigate their hyperactivation. Following that aim, Saliba et al. synthesized a new antagonist for the orphan G-protein coupled receptor 55, which decreased the production of prostaglandin E_2_ and reduced the activation of pro-inflammatory signalling pathways [3]. In line with this, other receptors regulating microglia function are being explored. This is the case for some purinergic receptors such as P2Y receptors, whose actions on microglia and neuroinflammation were elegantly reviewed by Gómez Morillas et al. [4]. These data highlight that the isolation of new receptors that can be pharmaceutically targeted might be relevant for treating neuroinflammatory disorders. Actually, a similar approach is followed for other immune-mediated pathologies, in which the inhibition of various receptors such as sodium-glucose co-transporter 2 [5], angiotensin receptor [6], or toll-like receptors [7] is being tested for controlling the exacerbated inflammatory response during acute kidney injury, COVID-19 infection, heart failure, etc.

Focusing on autoimmune responses, Amend et al. [8] and Carvalheiro et al. [9] investigated the effects of IL-10 and angiopoietin-2 in the context of lupus and systemic sclerosis, respectively. The former shed light on the poorly understood role played by IL-10 in the development of lupus, revealing that IL-10 outcome depends on the complex interplay among the different immune cells and the inflammatory microenvironment. The latter investigated, for the first time, how angiopoietin-2 can increase the production of pro-inflammatory mediators by monocytes from systemic sclerosis patients, which could contribute to the development of fibrotic processes in the skin of those patients. Fibrosis is also a key feature in chronic kidney injury, and Leong et al. [10] functionally demonstrated in experimental acute kidney injury mouse models that cyclophilin inhibition protects from renal injury and fibrosis, an observation that is accompanied by reduced innate immune cell infiltration.

Inflammation significantly contributes to skin malignancies, as reviewed by Ansary et al. [11] and Razib Hossain et al. [12]. Interestingly, Yang et al. [13] demonstrated in vitro and in vivo the therapeutic potential of the natural small molecule neferine as an anti-inflammatory drug for atopic dermatitis. Moreover, Hathaway-Schrader et al. [14] showed new insights for a better comprehension of the immune evasion in melanoma cells.

Historically, inflammation-related research mainly comprised studies related to immune cells and inflammatory mediators. However, in recent years, more multidisciplinary and integrative approaches have been applied for the dissection of the inflammatory process [15]. This is the case for the article by Weiss et al. [16], which showed an interesting view of the participation of histone deacetylases in the activation of pro-inflammatory signalling cascades and mediators in cultured macrophages. Moreover, another work explored the methylation status of specific promoters for their participation in neuronal inflammatory diseases. Hypermethylated O^6^-methylguanine-DNA methyltransferase is commonly observed in brain tumours. However, Teuber-Hanselmann et al. [17] reported an original hypermethylation of that gene in inflammatory conditions affecting the central nervous system. Likewise, the participation of adipokines and obesity in regulating inflammation and cardiovascular disease [18,19] and the impact of the YAP/TAZ signalling in the immunomodulatory responses of tumours [20] have also been discussed. Of note, Götz et al. [21] demonstrated in vivo the relevance of the complement system in the recruitment of neutrophils and M2-polarised macrophages to ischemic tissues, which, most likely, are the most relevant players in the observed improvement of angiogenesis.

The impact of environmental factors such as pollutants, microparticles, or endocrine disruptors is gaining interest. The latter is not only relevant for their interference with the normal function of the endocrine system, but endocrine disruptors can also participate in the pathophysiology of non-alcoholic fatty liver disease (NAFLD) (as reviewed by Cano et al.) by modulating liver metabolism [22]. In the same way, the food additive titanium dioxide worsened experimental colitis in mice carrying IBD genetic risk mutations [23], suggesting that the ingestion of industrial compounds can be detrimental to patients with IBD with an increased genetic predisposition. Silicon dioxide is well-known for causing lung silicosis, and novel pathophysiological mechanisms and treatment options were reviewed by Adamcakova et al. [24]. Interestingly, Wang et al. [25] demonstrated the efficacy of a novel class of pharmacological compounds, namely SUL-151, in decreasing neutrophilia after cigarette smoke exposure in mice.

This Special Issue also contains translational studies demonstrating the association between high concentrations of IL-6 receptor antagonist and reduced carbohydrate disorders and NAFLD progression in obese individuals [26], the impairment of circulating monocytes and the aberrant production of pro-inflammatory mediators in spinal cord injury patients [27], and the existence of a fibroinflammatory signature in human follicular fluid of the female reproductive system [28]. Furthermore, Di Paola et al. [29] showed the effects of the thrombopoietin receptor agonist Eltrombopag in the differentiation of macrophages obtained from children with immune thrombocytopenia. Altogether, these data show, in the human setting, how inflammation can also be at play in conditions not typically recognised as immune-mediated diseases.

Finally, in this compilation of articles, we find very interesting reviews updating a wide variety of topics such as hypoalbuminemia as a surrogate of infections [30], the resolution of inflammation in IBD or pain [31,32], the impact of inflammation in liver tumorigenesis and alcohol disease [33,34], and nutraceutical supplementation in obesity-associated disorders [35]. Moreover, Rafael-Vidal et al. reviewed the potential use of calcineurin inhibitors to treat lupus nephritis [36], and the molecular and cellular mechanisms regulating osteoporosis after spinal cord injury were described by Shams et al. [37].

We hope the lectors will appreciate reading these papers. We thank all the authors and reviewers for their dedication and proactive participation that made the realisation of this remarkable Special Issue possible.

## References

[1] Zhukovsky N., Silvano M., Filloux T., Gonzalez S., Krause K.-H. (2022). Alpha-1 Antitrypsin Reduces Disease Progression in a Mouse Model of Charcot-Marie-Tooth Type 1A: A Role for Decreased Inflammation and ADAM-17 Inhibition. Int. J. Mol. Sci..

[2] Rojas M., Ariza D., Ortega Á., Riaño-Garzón M.E., Chávez-Castillo M., Pérez J.L., Cudris-Torres L., Bautista M.J., Medina-Ortiz O., Rojas-Quintero J. (2022). Electroconvulsive Therapy in Psychiatric Disorders: A Narrative Review Exploring Neuroendocrine–Immune Therapeutic Mechanisms and Clinical Implications. Int. J. Mol. Sci..

[3] Saliba S.W., Gläser F., Deckers A., Keil A., Hurrle T., Apweiler M., Ferver F., Volz N., Endres D., Bräse S. (2021). Effects of a Novel GPR55 Antagonist on the Arachidonic Acid Cascade in LPS-Activated Primary Microglial Cells. Int. J. Mol. Sci..

[4] Gómez Morillas A., Besson V.C., Lerouet D. (2021). Microglia and Neuroinflammation: What Place for P2RY12?. Int. J. Mol. Sci..

[5] Feijóo-Bandín S., Aragón-Herrera A., Otero-Santiago M., Anido-Varela L., Moraña-Fernández S., Tarazón E., Roselló-Lletí E., Portolés M., Gualillo O., González-Juanatey J.R. (2022). Role of Sodium-Glucose Co-Transporter 2 Inhibitors in the Regulation of Inflammatory Processes in Animal Models. Int. J. Mol. Sci..

[6] Bellis A., Mauro C., Barbato E., Trimarco B., Morisco C. (2020). The Rationale for Angiotensin Receptor Neprilysin Inhibitors in a Multi-Targeted Therapeutic Approach to COVID-19. Int. J. Mol. Sci..

[7] Vázquez-Carballo C., Guerrero-Hue M., García-Caballero C., Rayego-Mateos S., Opazo-Ríos L., Morgado-Pascual J.L., Herencia-Bellido C., Vallejo-Mudarra M., Cortegano I., Gaspar M.L. (2021). Toll-Like Receptors in Acute Kidney Injury. Int. J. Mol. Sci..

[8] Amend A., Wickli N., Schäfer A.-L., Sprenger D.T.L., Manz R.A., Voll R.E., Chevalier N. (2021). Dual Role of Interleukin-10 in Murine NZB/W F1 Lupus. Int. J. Mol. Sci..

[9] Carvalheiro T., Lopes A.P., van der Kroef M., Malvar-Fernandez B., Rafael-Vidal C., Hinrichs A.C., Servaas N.H., Bonte-Mineur F., Kok M.R., Beretta L. (2020). Angiopoietin-2 Promotes Inflammatory Activation in Monocytes of Systemic Sclerosis Patients. Int. J. Mol. Sci..

[10] Leong K.G., Ozols E., Kanellis J., Badal S.S., Liles J.T., Nikolic-Paterson D.J., Ma F.Y. (2020). Cyclophilin Inhibition Protects Against Experimental Acute Kidney Injury and Renal Interstitial Fibrosis. Int. J. Mol. Sci..

[11] Ansary T.M., Hossain M.R., Kamiya K., Komine M., Ohtsuki M. (2021). Inflammatory Molecules Associated with Ultraviolet Radiation-Mediated Skin Aging. Int. J. Mol. Sci..

[12] Hossain M.R., Ansary T.M., Komine M., Ohtsuki M. (2021). Diversified Stimuli-Induced Inflammatory Pathways Cause Skin Pigmentation. Int. J. Mol. Sci..

[13] Yang C.-C., Hung Y.-L., Ko W.-C., Tsai Y.-J., Chang J.-F., Liang C.-W., Chang D.-C., Hung C.-F. (2021). Effect of Neferine on DNCB-Induced Atopic Dermatitis in HaCaT Cells and BALB/c Mice. Int. J. Mol. Sci..

[14] Hathaway-Schrader J.D., Norton D., Hastings K., Doonan B.P., Fritz S.T., Bethard J.R., Blum J.S., Haque A. (2022). GILT Expression in Human Melanoma Cells Enhances Generation of Antigenic Peptides for HLA Class II-Mediated Immune Recognition. Int. J. Mol. Sci..

[15] Panpetch W., Phuengmaung P., Hiengrach P., Issara-Amphorn J., Cheibchalard T., Somboonna N., Tumwasorn S., Leelahavanichkul A. (2022). Candida Worsens Klebsiella pneumoniae Induced-Sepsis in a Mouse Model with Low Dose Dextran Sulfate Solution through Gut Dysbiosis and Enhanced Inflammation. Int. J. Mol. Sci..

[16] Weiss U., Möller M., Husseini S.A., Manderscheid C., Häusler J., Geisslinger G., Niederberger E. (2020). Inhibition of HDAC Enzymes Contributes to Differential Expression of Pro-Inflammatory Proteins in the TLR-4 Signaling Cascade. Int. J. Mol. Sci..

[17] Teuber-Hanselmann S., Worm K., Macha N., Junker A. (2021). MGMT-Methylation in Non-Neoplastic Diseases of the Central Nervous System. Int. J. Mol. Sci..

[18] Feijóo-Bandín S., Aragón-Herrera A., Moraña-Fernández S., Anido-Varela L., Tarazón E., Roselló-Lletí E., Portolés M., Moscoso I., Gualillo O., González-Juanatey J.R. (2020). Adipokines and Inflammation: Focus on Cardiovascular Diseases. Int. J. Mol. Sci..

[19] Battineni G., Sagaro G.G., Chintalapudi N., Amenta F., Tomassoni D., Tayebati S.K. (2021). Impact of Obesity-Induced Inflammation on Cardiovascular Diseases (CVD). Int. J. Mol. Sci..

[20] Ortega Á., Vera I., Diaz M., Navarro C., Rojas M., Torres W., Parra H., Salazar J., De Sanctis J., Bermúdez V. (2021). The YAP/TAZ Signaling Pathway in the Tumor Microenvironment and Carcinogenesis: Current Knowledge and Therapeutic Promises. Int. J. Mol. Sci..

[21] Götz P., Braumandl A., Kübler M., Kumaraswami K., Ishikawa-Ankerhold H., Lasch M., Deindl E. (2021). C3 Deficiency Leads to Increased Angiogenesis and Elevated Pro-Angiogenic Leukocyte Recruitment in Ischemic Muscle Tissue. Int. J. Mol. Sci..

[22] Cano R., Pérez J.L., Dávila L.A., Ortega Á., Gómez Y., Valero-Cedeño N.J., Parra H., Manzano A., Véliz Castro T.I., Albornoz M.P.D. (2021). Role of Endocrine-Disrupting Chemicals in the Pathogenesis of Non-Alcoholic Fatty Liver Disease: A Comprehensive Review. Int. J. Mol. Sci..

[23] Conde J., Schwarzfischer M., Katkeviciute E., Häfliger J., Niechcial A., Brillant N., Manzini R., Bäbler K., Atrott K., Lang S. (2021). Titanium Dioxide Presents a Different Profile in Dextran Sodium Sulphate-Induced Experimental Colitis in Mice Lacking the IBD Risk Gene Ptpn2 in Myeloid Cells. Int. J. Mol. Sci..

[24] Adamcakova J., Mokra D. (2021). New Insights into Pathomechanisms and Treatment Possibilities for Lung Silicosis. Int. J. Mol. Sci..

[25] Wang L., Pelgrim C.E., Swart D.H., Krenning G., van der Graaf A.C., Kraneveld A.D., Leusink-Muis T., van Ark I., Garssen J., Folkerts G. (2021). SUL-151 Decreases Airway Neutrophilia as a Prophylactic and Therapeutic Treatment in Mice after Cigarette Smoke Exposure. Int. J. Mol. Sci..

[26] Skuratovskaia D., Komar A., Vulf M., Quang H.V., Shunkin E., Volkova L., Gazatova N., Zatolokin P., Litvinova L. (2021). IL-6 Reduces Mitochondrial Replication, and IL-6 Receptors Reduce Chronic Inflammation in NAFLD and Type 2 Diabetes. Int. J. Mol. Sci..

[27] Diaz D., Lopez-Dolado E., Haro S., Monserrat J., Martinez-Alonso C., Balomeros D., Albillos A., Alvarez-Mon M. (2021). Systemic Inflammation and the Breakdown of Intestinal Homeostasis Are Key Events in Chronic Spinal Cord Injury Patients. Int. J. Mol. Sci..

[28] Machlin J.H., Barishansky S.J., Kelsh J., Larmore M.J., Johnson B.W., Pritchard M.T., Pavone M.E., Duncan F.E. (2021). Fibroinflammatory Signatures Increase with Age in the Human Ovary and Follicular Fluid. Int. J. Mol. Sci..

[29] Di Paola A., Palumbo G., Merli P., Argenziano M., Tortora C., Strocchio L., Roberti D., Santoro C., Perrotta S., Rossi F. (2020). Effects of Eltrombopag on In Vitro Macrophage Polarization in Pediatric Immune Thrombocytopenia. Int. J. Mol. Sci..

[30] Wiedermann C.J. (2021). Hypoalbuminemia as Surrogate and Culprit of Infections. Int. J. Mol. Sci..

[31] Camba-Gómez M., Gualillo O., Conde-Aranda J. (2021). New Perspectives in the Study of Intestinal Inflammation: Focus on the Resolution of Inflammation. Int. J. Mol. Sci..

[32] Chávez-Castillo M., Ortega Á., Cudris-Torres L., Duran P., Rojas M., Manzano A., Garrido B., Salazar J., Silva A., Rojas-Gomez D.M. (2021). Specialized Pro-Resolving Lipid Mediators: The Future of Chronic Pain Therapy?. Int. J. Mol. Sci..

[33] Nagai N., Kudo Y., Aki D., Nakagawa H., Taniguchi K. (2021). Immunomodulation by Inflammation during Liver and Gastrointestinal Tumorigenesis and Aging. Int. J. Mol. Sci..

[34] Nowak A.J., Relja B. (2020). The Impact of Acute or Chronic Alcohol Intake on the NF-κB Signaling Pathway in Alcohol-Related Liver Disease. Int. J. Mol. Sci..

[35] Scarano F., Gliozzi M., Zito M.C., Guarnieri L., Carresi C., Macrì R., Nucera S., Scicchitano M., Bosco F., Ruga S. (2021). Potential of Nutraceutical Supplementation in the Modulation of White and Brown Fat Tissues in Obesity-Associated Disorders: Role of Inflammatory Signalling. Int. J. Mol. Sci..

[36] Rafael-Vidal C., Altabás I., Pérez N., Mourino Rodríguez C., Pego-Reigosa J.M., Garcia S. (2021). Calcineurin and Systemic Lupus Erythematosus: The Rationale for Using Calcineurin Inhibitors in the Treatment of Lupus Nephritis. Int. J. Mol. Sci..

[37] Shams R., Drasites K.P., Zaman V., Matzelle D., Shields D.C., Garner D.P., Sole C.J., Haque A., Banik N.L. (2021). The Pathophysiology of Osteoporosis after Spinal Cord Injury. Int. J. Mol. Sci..

